# Robot-assisted radical cystectomy with the clinical application of “Y-shaped” end-to-side ureteral anastomosis in elderly and obese patients

**DOI:** 10.1186/s12894-024-01684-5

**Published:** 2025-03-20

**Authors:** Yixuan Mou, Yeqing Mao, Zhenghong Liu, Pu Zhang, Yunkai Yang, Xiaolong Qi, Dahong Zhang, Qijun Wo

**Affiliations:** 1 Urology and Nephrology Center, Department of Urology, Affiliated People’s Hospital, Zhejiang Provincial People’s Hospital, Hangzhou Medical College, Hangzhou, China; 2 Urology Department of Urology, The First Affiliated Hospital of Zhejiang University, Hangzhou, Zhejiang China

**Keywords:** Bladder cancer, Urinary diversion, Radical cystectomy, End-to-side anastomosis, Cutaneous ureterostomy

## Abstract

**Objective:**

To investigate the clinical effect and safety of “Y-shaped” end-to-side ureteral anastomosis with robotic endoscopic technique in radical cystectomy (RC) and urinary diversion (UD) in elderly and obese patients with bladder cancer.

**Materials and methods:**

We retrospectively reviewed the records of 10 patients with bladder cancer who underwent robot-assisted laparoscopic radical cystectomy and “Y-shaped” end-to-side ureteral anastomosis under general anesthesia at Zhejiang Provincial People’s Hospital (Affiliated People’s Hospital, Hangzhou Medical College, Hangzhou, Zhejiang, China, 310014) from October 2018 to January 2021. Demographic and clinical data are summarized. The incidence of postoperative complications such as papillary retraction, ureteral stricture, anastomotic stenosis, anastomotic fistula and ureteral calculi were observed and analyzed.

**Results:**

A total of 10 elderly and obese patients successfully underwent RC with “Y-shaped” end-to-side ureteral anastomosis in this research. Median age was (80.6 ± 5.7) y and BMI was (25.12 ± 3.83) kg/m^2^. The operation time was (95 ± 26) min and the estimated intraoperative blood loss was (100.5 ± 35.6) ml, with no perioperative blood transfusion and no readmission 30 days after operation. No serious complications above Clavien-Dindo grade 4 occurred in the early (≤ 30 d) and late (> 30 d) after surgery. 1 patient developed fever three days after operation and was cured by strengthening anti-infection. 1 patient had a small amount of urine leakage at the anastomotic site after operation, and recovered after strengthening nutrition and maintaining the patency of abdominal drainage tube and single J tube. Postoperatively, the patients replaced the single J tube regularly and were followed up for 3–28 months (average 15 months) until April 10, 2021. In two cases, the ureterostomy nipple was slightly retracted and collapsed without special treatment. one case formed ureteral calculi and was treated conservatively. No ureteral stenosis, necrosis, anastomotic stenosis or severe anastomotic fistula, hernia around the stoma occurred. No visceral metastases or new lesions of urothelial carcinoma were observed. All patients were satisfied with the postoperative quality of life.

**Conclusions:**

The robot-assisted “Y-shaped” end-to-side ureteral anastomosis technique performed intracorporeally seems to be a straightforward, secure, and viable approach. It is considered suitable for radical resection of bladder cancer and urinary diversion in elderly and obese patients.

**Supplementary Information:**

The online version contains supplementary material available at 10.1186/s12894-024-01684-5.

## Introduction

Bladder cancer is a globally prevalent malignant tumor, ranking as the tenth most common cancer, with approximately 573,000 new cases annually [1]. The current clinical standard for managing localized invasive bladder cancer and non-muscle invasive bladder cancer refractory to Bacillus Calmette-Guérin (BCG) therapy is Radical Cystectomy (RC), followed by Urinary Diversion (UD) [2]. In recent years, there has been a significant shift towards minimally invasive approaches in surgical oncology, with robot-assisted radical cystectomy (RARC) emerging as a promising alternative to open surgery [3]. Comparative analyses have demonstrated that RARC exhibits superior perioperative safety and comparable oncological outcomes when contrasted with laparoscopic techniques [4, 5], and more recent research has begun to elucidate its long-term oncologic outcomes and patient-reported quality of life measures. A comprehensive meta-analysis conducted by Cella et al. has substantiated the position of robotic-assisted surgery as a favorable option, characterized by improved perioperative outcomes and equivalent oncological efficacy. However, despite these advancements, there is an ongoing necessity for rigorous evaluation and refinement of surgical techniques to maximize patient outcomes [6]. Various methods of urinary diversion, such as the in situ ileocecal neobladder and Bricker ileum bladder, often necessitate the use of intestinal segments. However, intracorporeal urine diversion can lead to complications like enterocutaneous fistula [7, 8] and ureter-enteric anastomotic stenosis [9], Challenges like insufficient bowel conditions, advanced age, obesity, underlying diseases, patient preferences, and deficiencies in stoma care limit the application of these diversion methods. Data from recent studies suggest that a minority of patients undergoing RARC (3%) select totally intracorporeal urinary diversion [10].

Cutaneous ureterostomy (CU) has demonstrated superiority in terms of operative time, perioperative risks, and anesthetic risks, establishing itself as a critical modality for UD [11, 12]. The enhanced capabilities of robotic surgical systems, enjoying a highly magnified view with high resolution, well-suited for meticulous, and it improves success rate of CU in cystectomy by reducing operative difficulty. The Department of Urology at Zhejiang Provincial People’s Hospital has successfully performed 10 cases of robot-assisted laparoscopic radical cystectomy for bladder cancer with unilateral abdominal wall stoma of bilateral ureters, utilizing the “Y-shaped” end-side anastomosis of the ureter. Postoperative follow-up ranging from 3 to 28 months has indicated satisfactory initial results.

## Materials and methods

### Patient information

After obtaining approval from the Institutional Review Board, this study enrolled 10 bladder cancer patients admitted to Zhejiang Provincial People’s Hospital (Affiliated People’s Hospital, Hangzhou Medical College, Hangzhou, Zhejiang, China, 310014) from October 2018 to January 2021. The patient selection was based on predefined inclusion and exclusion criteria, comprising eight males and two females.

The inclusion criteria were as follows: ①bladder cancer patients with a Body Mass Index (BMI) of ≥ 23 and an age of ≥ 75 years; ② the surgical treatment method was a “Y-shaped” end-to-side ureteral anastomosis; ③ a postoperative follow-up period of ≥ 3 months; ④ regular postoperative checkups conducted at Zhejiang Provincial People’s Hospital; ⑤ normal communication skills. The exclusion criteria included: ① patients with bladder cancer, with a BMI < 23 or age < 75 years, or those who did not undergo a “Y-shaped” end-to-side ureteral anastomosis; ② patients with severe organ dysfunction or poor baseline health, rendering them unfit for surgical intervention; ③ patients with an insufficient follow-up duration; ④ patients lacking complete clinical data. Age classification adhered to the World Health Organization’s (WHO) definition, categorizing individuals aged 60–74 as young elderly and those aged 75–89 as elderly [13]. Obesity was defined based on the WHO’s classification for adult Asians, with a Body Mass Index (BMI) of ≥ 23 being categorized as obesity [14]. Prior to RARC, all patients underwent comprehensive preoperative assessment, including pulmonary computed tomography (CT), ultrasound (cardiovascular and abdominal), electrocardiogram, preoperative blood test, CT urography, and cystoscopy with biopsy or trans-urethral resection of a bladder tumor (TURBT).

### Surgery procedures

All patients underwent robot-assisted laparoscopic radical cystectomy + “Y-shaped” end-to-side ureteral anastomosis (two cases underwent obturator lymphatic biopsy, while the remaining eight cases did not undergo pelvic lymph node dissection or biopsy. In female cases, the uterus and ovaries were preserved). The surgical video is available in the additional material.

### Stage 1: port placement

**Fig. 1:**
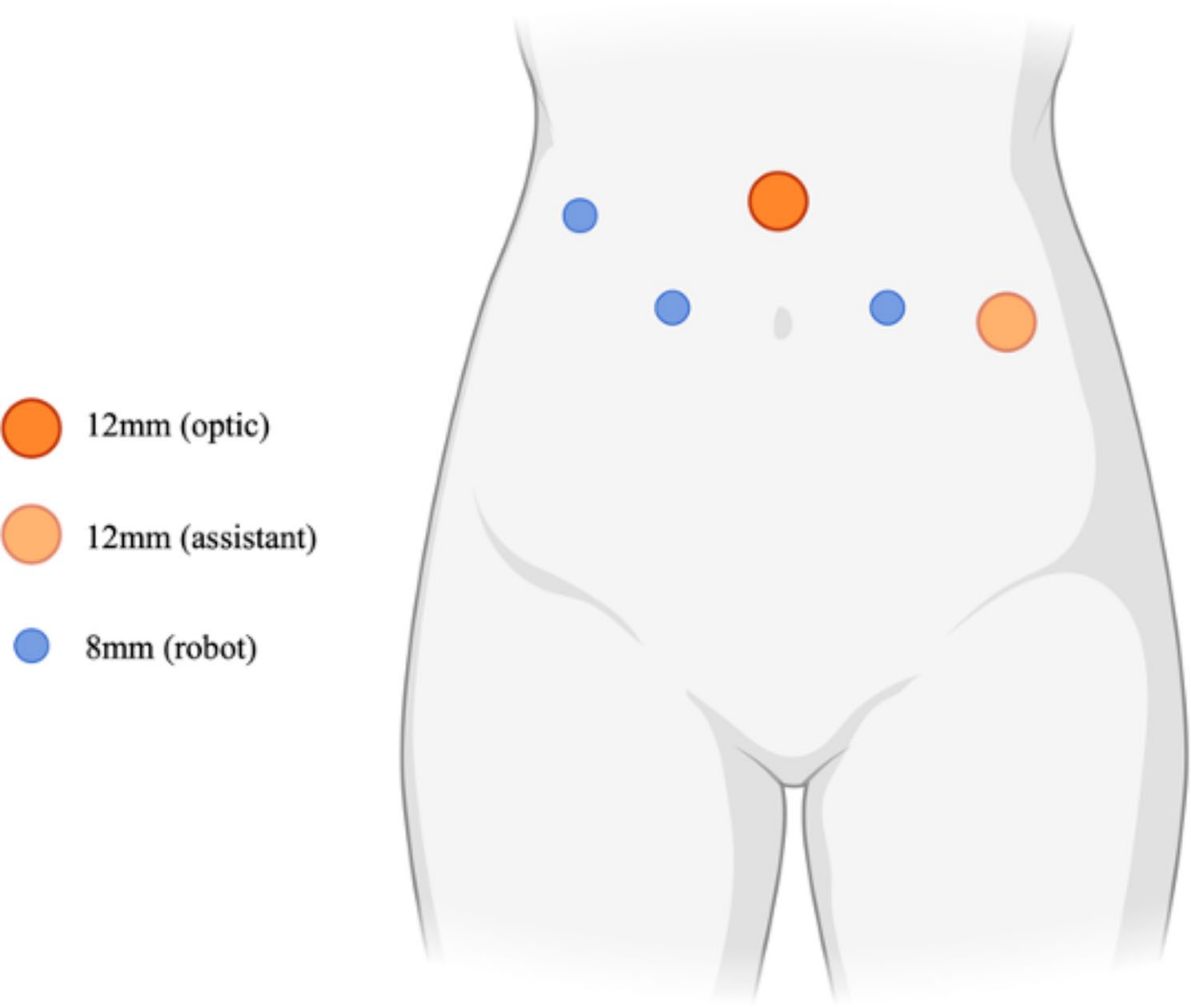
Port placement of robot-assisted laparoscopic radical cystectomy

Patients were positioned in 30° Trendelenburg position post general anesthesia and intubation, followed by catheterization and preparation. The 4-arm da Vinci Si system Intuitive Surgical from the American company was utilized. A 12-mm Trocar for the camera was positioned 2 cm above the umbilicus, with a 30° lens. Three robotic 8-mm Trocars and an additional 12-mm Trocar were placed under direct vision, with assistant ports on the left abdominal wall (as shown in Fig. 1). The robotic arms were then equipped as follows: the first with unipolar curved electric scissors, the second with Maryland bipolar forceps, and the third with window-hole grasping forceps. Pneumoperitoneum was maintained at 10 mmHg.

### Stage 2: dual ureteral unilateral dermatostomy with “Y-shaped” ureteral end-to-side anastomosis technique

**Fig. 2:**
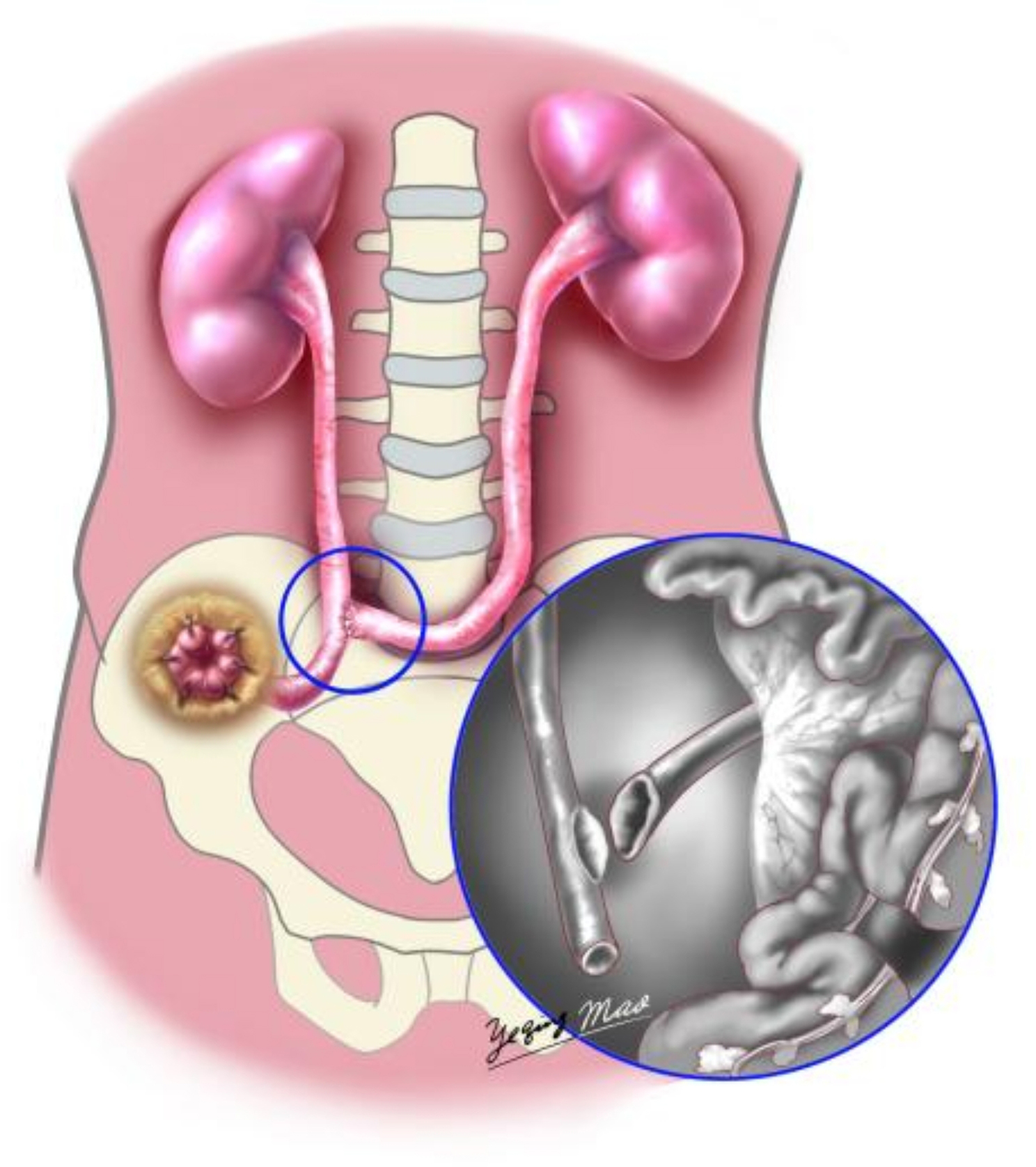
Diagrammatic sketch of “Y-shaped” end-to-side ureteral anastomosis

**Fig. 3:**
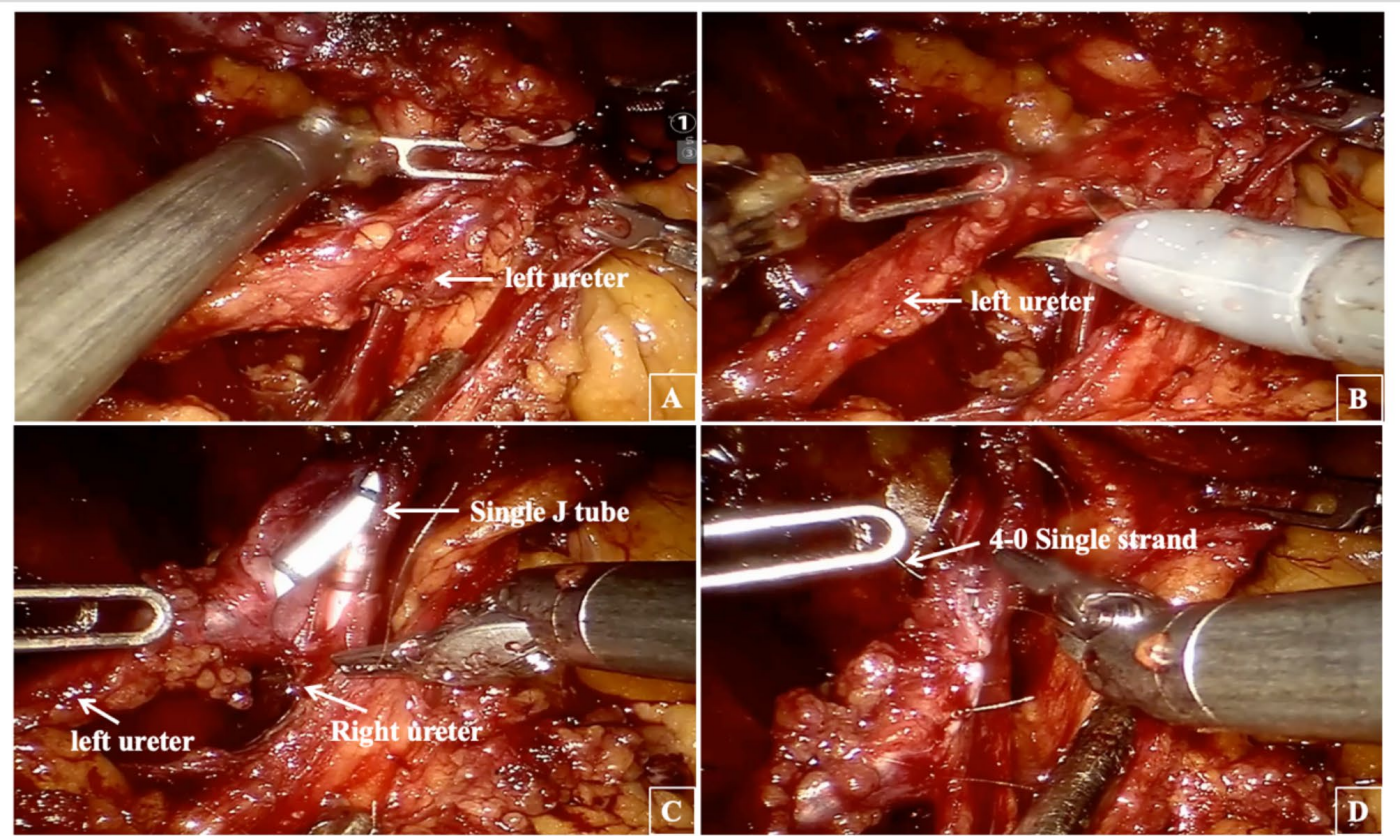
Operation procedure. Pull the left ureter to the contralateral side (**A**); Longitudinal incision of left ureteral opening about 1.5 cm for anastomosis (**B**); A single J tube was placed upward from the right ureteral end into the renal pelvic cavity (**C**); A 4 − 0 Single strand absorbable line was used for “Y-shaped” end-to-side ureteral anastomosis (**D**)

After total cystectomy, the right ureter was dissociated low for approximately 15 cm and ligated with a hem-o-lock clip, the left ureter was managed similarly, ensuring negative margins on both ureteral stumps confirmed by frozen section. After dissociating the non-avascular area in the sigmoid mesentery, the left ureter was rerouted to anastomose with the right ureter without tension. The end of the right ureter was pulled out of the abdominal wall, prelabeled by the Stoma nurse, and properly fixed. Both ureters were incised longitudinally 1.5 cm, and the right ureter was lengthwise cut about 1.5 cm at 10 cm from the broken end of the right ureter. End-to-side anastomosis was performed using a 4 − 0 absorbable line to form a “Y” shape (as shown in Figs. 2 and 3). A single J tube was inserted into the right ureteral end and directed into the renal pelvis before anastomosis completion.

### Stage 3: abdominal cavity closure

After confirming no urine leakage at the anastomosis site. The peritoneum on the right side was closed, and the ureters were placed outside the peritoneum (peritonealization). A pelvic drainage tube was inserted. A median incision was made to extract the radical cystectomy specimen. After removing the robotic arms and trocars, the right ureteral orifice was sutured and fixed to the right abdominal wall.

### Endpoints

The primary endpoints of this study encompass the surgical success rate, operative duration, intraoperative blood loss, blood transfusions. Additionally, we will monitor the length of hospital stay, as well as the duration of drainage tube and single-J ureteral stent placement. The secondary endpoints focus on the incidence of postoperative complications, which include papillary retraction, ureteral stricture, anastomotic stenosis, anastomotic fistula, and ureteral calculi. These complications will be assessed at 3-day intervals initially, followed by monthly evaluations postoperatively. Follow-up time > 3 months.

## Results

Surgeries were successful in all 10 cases. All patients included in this study had an average age of (80.6 ± 5.7) years and a BMI of (25.12 ± 3.83) kg/m². The median age-corrected Charlson comorbidity index (ACCI) was eight, and the median American Society of Anesthesiologists (ASA) score was three. Clinical staging revealed T_1G3_ in one case and T_2_in nine cases. None of the patients had a history of external radiotherapy or intravenous chemotherapy, and there was no evidence of systemic organ metastasis. Additionally, none of the patients had undergone traditional open abdominal surgery before RARC.

Among the patients, three cases had impaired heart function (EF < 50%), seven cases had hypertension, six cases had diabetes mellitus (two cases of type 1, four cases of type 2), five cases had chronic lung disease, and three cases had anemia. The average operation time was (95 ± 26) minutes. The estimated intraoperative blood loss was (100.5 ± 35.6) ml, with no perioperative blood transfusion required, and there were no readmissions within 30 days after the operation. No serious complications above Clavien-Dindo grade 4 occurred in the early (≤ 30 days) and late (> 30 days) postoperative periods.

Postoperative ureteral stricture may cause difficulty in inserting ureteral stents again or require renal puncture drainage. In this study, three patients voluntarily chosed to have their ureteral stents placed for life, with a replacement frequency of three months. The extraction time of single-J tube in the remaining seven patients was (69.86 ± 9.8) days. One case developed fever 3 days after the operation, which resolved with enhanced anti-infective therapy. Another case experienced minor urine leakage at the anastomotic site, which resolved with improved nutrition and ensuring the patency of the abdominal drainage tube and single J ureteral stent. Postoperative follow-up, which lasted an average of 15 ± 4.5 months (median 12 months) until April 10, 2021, included chest and abdominal CT scans and/or routine blood and biochemical tests at 1–3 month intervals. In two cases, slight retraction of the ureterostomy nipple occurred without necessitating specific treatment. One case developed ureteral calculi, which were managed conservatively. No ureteral stenosis, necrosis, anastomotic stenosis, severe anastomotic fistula, or hernia around the stoma were found. No visceral metastases or new lesions of urothelial carcinoma were observed. All patients were satisfied with the postoperative quality of life.

## Discussion

The International Consultative Committee on Urological Diseases (ICUD-EAU) conducted a comprehensive meta-analysis of 16,000 postoperative urinary diversion patients with bladder cancer from 11 medical centers. The findings from this extensive study have highlighted the prevalence of different urinary diversion methods used in clinical practice. Specifically, the ileal outflow tract was utilized in 42.2% of the cases, the in situ cystectomy method was employed in 38% of the cases, and ureteral dermatostomy was performed in 10.4% of the cases.

In situ ileal neobladder and ileal outflow tract procedures need exquisite surgical techniques and long operative time [15, 16]. The concept of enhanced recovery after surgery (ERAS) is well established, with the goal of facilitating a quicker postoperative recovery for patients. However, this approach is limited by the condition of the intestinal tract and the patient’s overall fitness. It is crucial that the intestinal segments used for the reconstruction of the neobladder and ileal outflow tract are in a healthy condition to avoid complications associated with the use of these tubes. Therefore, patients with conditions such as short bowel syndrome, inflammatory diseases of the small intestine, and those with a history of extensive radiation exposure to the ileum are typically not considered suitable candidates for these complex procedures.

Cutaneous ureterostomy is a relatively simple procedure with a short learning curve and operative time. It does not interfere with intestinal function and allows for quick postoperative recovery, making it particularly suitable for patients with a limited life expectancy, palliative total cystectomy, severe medical comorbidities, associated intestinal diseases, or for those who decline bowel-related operations (see Tables 1 and 2).

**Table 1:** Basic clinical data

	Cases
Gender	
Male	8
Female	2
History diseases	
Impaired heart function (EF < 50%)	3
Hypertension	7
Diabetes	6
Chronic lung disease	5
Anemia	3
Average age (X ± S years)	80.6 ± 5.7
BMI (X ± S kg/m2)	25.12 ± 3.83
Pathological	
Muscle invasive bladder cancer	9
Bladder adenocarcinoma	1
Positive ureteral margins	0

**Table 2:** Clinical efficacy

	Results
Operation time (X ± S minutes)	95 ± 26
Bleeding volume(X ± S ml)	100.5 ± 35.6
Postoperative hospitalization(X ± S days)	5 ± 1.54
Perioperative blood transfusion (cases)	0
Duration time of drainage tube(X ± S days)	3 ± 2.57
Extraction time of single-J tube(X ± S days)	69.86 ± 9.8
Followed up time (months)	15 ± 4.500
Postoperative complication (cases)	
Ureteral calculi	0
Reflux / Ureteral obstruction	0
Ureterostomy nipple retracted	2
Ureteral stenosis	0
Anastomotic stenosis	0
Anastomotic fistula Anastomotic hernia around the stoma	0 0
Tumor recurrence /metastasis	0

However, CU comes with certain drawbacks, including the potential for ureteral strictures [17], necrosis, and the often necessary implementation of bilateral ureterostomy, which can complicate postoperative care. Excessive or insufficient dissociation of the ureter during the procedure may lead to high ureteral tension, resulting in impaired blood supply to the ureter. To enhance surgical quality and mitigate these challenges, it is crucial to adhere to some basic principles: ensuring the blood supply of the ureter (appropriate length of ureteral dissection, reducing the tension of the ureteral outflow tract), attempting to achieve unilateral ureterostomy and so on.

In order to realize the above basic principles, the Department of Urology in Zhejiang Provincial People’s Hospital has leveraged previous reports and experience of laparoscopic and Da Vinci robotic laparoscopic surgery, explored the whole endoscopy operation, fine ureteral dissociating technology assisted by robots, successfully released the Robot-assisted “Y-shaped” end-to-side ureteral anastomosis technique under whole endoscopy for radical cystectomy, with good recovery and low complication rates. With innovations in surgical techniques, both continent and incontinent diversions are available for urinary reconstruction after RC [18]. However, the Bricker ileal conduit procedure or in situ ileal neocystectomy requires the well physical fundamentals of patients [19–21], and the long duration of the procedure, intestinal complications, anastomotic stenosis, and other factors that may adversely affect the prognosis of elderly and obese patients. To avoid the difficulties in stoma care and reduce the costs with bilateral stoma, there is a burgeoning interest in unilateral stoma methods, such as end-to-end pyeloureterostomy ureteral anastomosis, single umbilical stoma, etc. [22, 23]. It has been definitively demonstrated that end-to-side ureteral anastomosis has lower urological complications in renal transplantation [22], and low tension of intraoperative anastomosis can effectively prevent stenosis, anastomotic stenosis and severe anastomotic fistula. Compared to bilateral stoma, Bricker ileal conduit procedure or in situ ileal neocystectomy, the simplicity of stoma care, reduced hospital stay, and diminished complication rates contribute significantly to lower treatment costs. Furthermore, this procedure offers a shorter learning curve for clinical practice.

We believe that the technical points and advantages of this procedure assisted by Da Vinci robot include: (1) The robotic surgical system affords a high-definition operative view, enhancing visual acuity during surgery. (2) Minimize the length of ureteral dissociation, thereby conserving the length and vascular integrity of the distal ureter, and precluding unnecessary manipulation that could compromise ureteral perfusion. A strategic approach, such as translocating the left ureter through the mesenteric non-avascular plane to the right side, has been effective. Our experience indicates that positioning the left ureter approximately 1 cm lateral to the superior aspect of the iliac vessels is typically sufficient. Likewise, the anastomotic site for the right ureter is often selected 1 cm lateral to the superior aspect of the right iliac vessel. (3) To preserve ureteral blood supply, the technique avoids ureteral torsion and dissects along the ureter’s longitudinal axis on the nonmembranous aspect. (4) The caliber of the “Y-shaped” end-to-side ureteral anastomosis should be appropriate, with a preference for 4 − 0 or 5 − 0 single-strand absorbable sutures to facilitate precise anastomosis. (5) Utilizing peritoneal coverage (peritonealization) over the ureter promotes healing in the anastomotic region, reducing the risk of anastomotic fluid leakage into the peritoneal cavity and preventing internal herniation. (6) Whole endoscopy operation of urinary diversion reduces the exposure time of the bowel. Although the present study does not offer a direct comparison between robot-assisted laparoscopic “Y-shaped” end-to-side ureteral anastomosis and open surgery, previous research has demonstrated the substantial benefits of robot-assisted surgery in terms of perioperative and functional outcomes [24, 25]. The minimally invasive nature of this approach can lead to reduced postoperative pain, decreased blood loss, and shortened hospital stays, all of which are especially pertinent to the care of elderly and obese patients.

Moreover, the manual dilation of the ureteral diameter, achieved by temporarily clamping the ureter with a hem-o-lock prior to anastomosis, facilitates the anastomotic process. However, this technique mandates vigilant postoperative monitoring of renal function to preempt any potential complications. Creta et al. [26] proposed that elderly patients with bladder cancer may experience deterioration of renal function due to acute kidney injury, after RC + UD. Considering the stoma habitus, all patients in this study had a right-sided stoma through pulling the left ureter to the right side with an end-to-side anastomosis. Nonetheless, the alternative approach of mobilizing the right ureter to the left side merits consideration. Based on our previous study, the use of the greater omentum to envelop the ureter or the anastomotic region to augment blood supply is a strategy that requires further investigation and analysis.

The management of single J tubes in our procedure warrants further investigation, taking into account variables such as the number of tubes, duration of indwelling time, postoperative replacement or removal, and the optimal diameter. In this study, the tailored placement of one or two single J tubes was determined based on the caliber of the right ureter. Given the prevalence of ureteral stenosis as a postoperative complication, routine changes of single J tubes under the guidance of an ultra-smooth guidewire were conducted during the postoperative period. While the accidental displacement of a single J tube could lead to challenges in stent tube repositioning, this was mitigated through thorough patient education on stoma care and secure stent tube fixation, preventing such occurrences in this study.

To enhance surgical safety and minimize operation time, routine or extended node dissection was not performed, and only two patients underwent obturator lymphatic biopsy. Considering the potential for uroepithelial carcinoma to present multisource lesions and the possibility of upper tract urothelial carcinoma (UTUC), we routinely performed intraoperative frozen section diagnosis of the ureteral stump to preclude the risk of residual urothelial carcinoma in the ureteral remnant.

Thus, choose the appropriate mode of urinary diversion individually is crucial. We summarized the target population or indications for performing “Y-shaped” end-to-side ureteral anastomosis: emphasis on preoperative prognosis and assessment, including the degree of obesity [26] and/or pelvic adiposity, economic ability, bowel conditions, objective quantitative indicators such as preoperative BMI, CT abdominal wall fat thickness, ureteral diameter can further evaluate. This method can become one of the important surgical strategies for elderly obese patients with a body mass index greater than 24 kg/m^2^. However, it is important to note that this study is a single-center, retrospective analysis, which may introduce selection bias. The small number of cases and the relatively short follow-up period mean that these findings require further validation through prospective, multicenter clinical trials with larger sample sizes to ensure broader applicability. Moving forward, we plan to extend our investigation to include long-term follow-up studies focusing on elderly obese patients who have undergone Y-shaped end-to-end ureteral anastomosis. Additionally, we aim to conduct prospective cohort studies with an expanded sample size to more comprehensively evaluate the long-term outcomes associated with this surgical technique.

## Conclusion

“Y-shaped” end-to-side ureteral anastomosis technique has significant application value under robot-assisted laparoscopic radical cystectomy + urinary diversion, demonstrates great therapeutic effects. Moreover, this procedure holds great significance in enhancing patients’ quality of life and reducing stoma costs.

## Electronic supplementary material

Below is the link to the electronic supplementary material.

Supplementary Material 1

## Data Availability

Data can be obtained from the corresponding author.
